# Nasal Exposure to Environmental PM2.5 Drives Testicular Pyroptosis and Tissue Damage: Evidence from Inhibitor Intervention and Multi Source Data

**DOI:** 10.21203/rs.3.rs-8701799/v1

**Published:** 2026-09-11

**Authors:** Luo Yi, Wang Cao, Ma Hong

**Affiliations:** 1/2: Zunyi City, Guizhou Province, Dalian Road, Zunyi Medical University Affiliated Hospital of Children ’s Chest Secretion Ward ; 563000

**Keywords:** PM2.5, Caspase-1, Pyroptosis, Transcriptome, PPI (Protein-Protein Interaction)

## Abstract

**Objective:**

Focusing on the process by which PM2.5 induces NLRP3/Caspase-1 axis activation and drives GSDMD-mediated pyroptosis, this study aimed to construct an integrated framework combining evidence from a PM2.5 animal model, intervention with a pyroptosis inhibitor, and reproducible screening of public transcriptomic databases.

**Methods:**

A total of 24 male Sprague-Dawley rats were randomly divided into four groups (n=6 per group): control (normal saline), Caspase-1 inhibitor Z-YVAD-FMK (5 μg/mg), PM2.5 (10 mg/kg), and PM2.5 + Caspase-1 inhibitor (PM2.5 exposure + Z-YVAD-FMK). After 4 weeks of exposure, mating tests were conducted followed by euthanasia. Testicular and epididymal tissues were collected for hematoxylin-eosin (HE) staining and sperm quality analysis. Key proteins associated with the pyroptotic response in testicular tissue were detected by Western Blot (WB). Concurrently, the public GEO dataset GSE189187 (GC-2spd cells, PM2.5-treated vs. control, 3 vs. 3) was analyzed for differential expression, followed by enrichment and Protein-Protein Interaction (PPI) network analyses. Hub genes were identified using the MCODE and CytoHubba plugins in Cytoscape software, and these network hubs were cross-validated with the evidence chain from the animal study.

**Results:**

Exposure to PM2.5 led to decreased sperm count and motility, along with a significant increase in sperm abnormality rate and a reduction in spermatogenic cell number. Western Blot analysis showed significantly elevated expression of the apoptosis-associated speck-like protein containing a CARD (ASC), Caspase-1, and Gasdermin D protein in the PM2.5 group. These changes were reversed by intervention with the Caspase-1 pyroptosis inhibitor. Transcriptomic differential analysis identified 217 significant differentially expressed genes (DEGs: 91 up-regulated, 126 down-regulated). Separate GO and KEGG enrichment analyses (Over-Representation Analysis, ORA) for up- and down-regulated genes revealed that DEGs were primarily enriched in antiviral/interferon-related immune processes. Pathways associated with tissue injury were also significantly enriched. Two pyroptosis-related DEGs were identified (Hmox1 up-regulated, Stat1 down-regulated). A PPI network constructed from these candidate genes was subjected to topological analysis in Cytoscape, and further screening with CytoHubba yielded 10 hub genes, suggesting that the PM2.5-related transcriptional response involves an interferon-STAT axis-driven inflammatory interaction network.

**Conclusion:**

PM2.5 from vehicle exhaust impairs male reproductive function. The pyroptosis inhibitor protects reproductive function by suppressing the NLRP3/Caspase-1 signaling pathway. Furthermore, PM2.5 can activate the differential expression of pyroptosis-related genes at the transcriptional level, thereby promoting an inflammatory interaction network that contributes to systemic inflammation.

## 1. Introduction

In recent years, male infertility has become a global concern, with declining sperm quality being a key pathogenic factor ^[1]^. The aggravation of atmospheric pollution has contributed to a yearly increase in male infertility rates, and PM2.5 from vehicle exhaust is considered a significant pollutant contributing to this issue. PM2.5 has a complex composition, posing substantial and potent pathogenic risks. Toxicological research on PM2.5 in humans has primarily focused on the respiratory, cardiovascular, and nervous systems, with relatively limited investigation into the male reproductive system ^[2]^. Epidemiological studies indicate that long-term exposure to airborne PM2.5 is closely associated with increased morbidity and mortality from respiratory and cardiovascular diseases^[3,4]^. Early-life PM2.5 exposure may affect the development of testicular tissue and have potential long-term impacts on the susceptibility to reproductive system diseases related to environmental PM2.5 exposure later in life ^[5]^. Furthermore, the molecular toxic mechanisms of PM2.5 involve oxidative stress, inflammatory responses, genotoxicity, and immunotoxicity^[6,7]^ . Therefore, investigating the underlying toxicological mechanisms linking PM2.5 to reproductive system damage is crucial.

Pyroptosis is a programmed inflammatory cell death driven by inflammasomes. In the canonical pathway, NLRP3 recruits ASC to activate caspase-1, which then cleaves GSDMD to form membrane pores and promotes the maturation and release of IL-18/IL-1β, leading to cell swelling, lysis, and amplification of inflammation^[8]^ . Previous studies in models such as the respiratory tract have demonstrated that PM2.5 can activate the NLRP3 inflammasome via upstream signals like ROS/NF-κB and trigger pyroptosis. However, experimental validation and reproducible omics evidence for the pathway linking PM2.5 → testicular tissue damage → caspase-1-dependent pyroptosis remain insufficient^[9]^.

In recent years, with the advancement of next-generation sequencing technologies, it has become possible to rapidly identify biological pathways and genes perturbed in toxicology through bioinformatic genomic analyses. These approaches effectively elucidate mechanisms and pathways, enabling high-throughput screening of toxicological relationships. The emergence of high-throughput sequencing has proven effective in elucidating pathogenic genes in various human diseases, aiding in the exploration of underlying toxic mechanisms ^[10]^. For instance, Cai et al. utilized the Gene Expression Omnibus (GEO) database to identify nine hub genes for polybrominated diphenyl ethers, which may play significant roles in their toxicological mechanisms^[11]^.

In this study, we employed a four-group animal intervention model incorporating a Caspase-1 inhibitor for causal verification and re-analyzed the gene expression profile from dataset GSE189187. Enrichment analyses of differentially expressed genes (DEGs) were performed using Gene Ontology (GO) and the Kyoto Encyclopedia of Genes and Genomes (KEGG) pathways. Hub genes within key modules were validated by comparing them with a series of changes observed in the animal intervention model. This work provides new directions for researching the hub genes involved in PM2.5-induced testicular pyroptosis mediated via the NLRP3/Caspase-1 signaling pathway following long-term exposure.

## 2. Materials and Methods

A batch of urban particulate matter (PM2.5) [Product No.: 1648a; Specification: 2G; Brand: NIST] was purchased from Dongguan Baishun Biotechnology Co., Ltd., on May 28, 2025. The particles were fully immersed in sterile normal saline and then subjected to four rounds of ultrasonication (40 minutes each) followed by filtration through sterile medical gauze. The suspension was subsequently centrifuged at 12,000 rpm for 30 min at 4°C. The resulting pellet was freeze-dried using a freeze-drier, and the obtained PM2.5 sample was stored at −80°C. Within 24 hours before use, a PM2.5 suspension of the desired concentration was prepared by adding sterile normal saline and agitating the mixture overnight at 37°C on a constant-temperature shaker at 60 rpm. The prepared PM2.5 suspension could be stored at 4°C for up to one week and was vortexed thoroughly before use.

Experimental animals were purchased from the Laboratory Animal Center of Zunyi Medical University [License No.: SYXK (Qian) 2021-0004]. This study was approved by the Experimental Animal Welfare and Ethics Committee of the participating institution (Approval No.: zyfy-an-2023-0013). A total of 24 specific pathogen-free (SPF) male Sprague-Dawley (SD) rats (4 weeks old, body weight 95 g ± 10 g) were used. The rats were housed individually in a barrier-system environment. They were randomly divided into four groups (n=6 per group): Group 1 served as the control group, receiving intranasal instillation of normal saline; Group 2 received the Caspase-1 inhibitor Z-YVAD-FMK (intraperitoneal injection, 5 μg/mg); Group 3 was exposed to PM2.5 via intranasal instillation (dose: 10 mg/kg); Group 4 received combined treatment with PM2.5 intranasal instillation and Z-YVAD-FMK intraperitoneal injection (intranasal instillation first, followed by intraperitoneal injection of Z-YVAD-FMK 30 minutes later). Z-YVAD-FMK is a cell-permeable, irreversible selective inhibitor of caspase-1 ^[12]^. The exposure protocol consisted of 5 days of treatment followed by 2 days of rest, repeated for a total duration of 4 weeks.

After the 4-week exposure period and a subsequent 2-day rest, the rats were weighed and euthanized via CO_2_ asphyxiation(1.5L/min/100g). Both testes and epididymides were then harvested and weighed. One testis from each animal was weighed to calculate the organ coefficient using the following formula: Organ Coefficient = (Organ Wet Weight [g] / Body Weight [g]) × 100%.

The contralateral testis and epididymis from each animal were collected for histopathological analysis. Specifically, testis tissues were rinsed with PBS, fixed in 10% paraformaldehyde for 24 hours, sagittally dissected and fixed for an additional 24 hours, and then embedded in paraffin. Sections were subsequently cut, deparaffinized, stained with Hematoxylin and Eosin (H&E), dehydrated, and cleared. A detailed evaluation of seminiferous tubule and spermatogenic cell morphology, sperm count, and the presence of abnormal sperm in the lumen was performed under an optical microscope (Leica, Germany, DM3000). Four random fields per animal were examined to compare differences among the four groups.

Western blotting was performed to detect the expression levels of ASC, caspase-1, GSDMD, and the internal reference protein β-Actin. Grayscale analysis was conducted using ImageJ software, with target protein bands normalized to β-Actin for statistical analysis. A p-value < 0.05 was considered statistically significant.

## 3. Data and Processing

The dataset was retrieved from the Gene Expression Omnibus (GEO, https://www.ncbi.nlm.nih.gov/geo/) database at NCBI using the keywords “PM2.5” and “GC-2spd cells”. The dataset GSE189187 met the search criteria. The PM2.5 used in the GSE189187 dataset for this analysis was derived from biomass combustion. Collected bulk fly ash was fractionated by a cyclone, and particles with an aerodynamic cutoff diameter of 2.5 μm were used for bioassays. The GSE189187 dataset contains a total of 6 samples, comprising 3 PM2.5-treated samples and 3 control samples.

The GSE189187 dataset (GC-2spd cells, PM2.5-treated vs. control, 3 vs. 3) was selected for differential expression screening, generation of a top gene heatmap, Over-Representation Analysis (ORA) for enrichment, and Protein-Protein Interaction (PPI) network analysis. Differential gene identification was performed using the statistical software RStudio and relevant packages for bioinformatic analysis between the PM2.5 and control groups. Given the small sample size (3 vs. 3), where the False Discovery Rate (FDR) after multiple correction might be overly conservative, a combination threshold of P.Value and ∣log2FC∣ was employed at the screening stage to obtain candidate gene sets, while the adj.P.Val was retained in tables as a robustness reference ^[13]^. Separate GO and KEGG ORA enrichment analyses were conducted for the screened up-regulated (DEG_up_screen) and down-regulated (DEG_down_screen) gene sets. A focused screening for pyroptosis-related genes was performed from the DEG_screen_all set, and the resulting Pyroptosis_DEGs were used as seed genes for subsequent network analysis ^[14]^.

These Pyroptosis_DEGs and other necessary candidate gene sets were then submitted to the STRING database (confidence score threshold set to 0.7) to construct a PPI interaction map. This PPI map was imported into Cytoscape for further analysis. The MCODE plugin was used to extract significant modules from the network, and the cytoHubba plugin was employed to rank and identify the top 10 hub genes. Data are presented as mean ± standard deviation. One-way analysis of variance (ANOVA) was performed in GraphPad Prism 9.0. Normality and homogeneity of variances were first assessed, followed by F-test and Tukey’s test for multiple comparisons. A p-value < 0.05 was set as the significance threshold. Statistical graphs were plotted using mean ± standard deviation.

The STRING online database was utilized to construct a Protein-Protein Interaction (PPI) network for key modules, with an interaction score > 0.4 considered significant for high-confidence interactions. Disconnected nodes were hidden in the network. Cytoscape software was used for PPI network visualization. Furthermore, the MCODE plugin was applied to identify significant modules within the PPI network^[15,16]^

## 4. Results

Following standardized processing of the GSE189187 dataset, the results are shown in Figure 1. PCA analysis revealed distinct gene expression profiles between the PM2.5 and control groups. Applying the screening criteria (adjusted p-value < 0.05, ∣log_2_FC∣ > 1) yielded 217 differentially expressed genes (DEGs), consisting of 91 up-regulated and 126 down-regulated genes.

As shown in Table 1, the testes from the model rats were weighed after tissue collection. Compared with the Control group, no significant changes were observed in body weight and testicular weight in the pyroptosis inhibitor group (p > 0.05). Both the PM2.5 group and the PM2.5 + pyroptosis inhibitor group exhibited a statistically significant reduction in body and testicular weight (p < 0.01). Furthermore, a significant difference was also observed between the PM2.5 group and the PM2.5 + pyroptosis inhibitor group (p < 0.05). These data indicate that PM2.5 exposure led to a statistically significant decrease in body and testicular weight (p < 0.05).

HE staining results (Figure 2) demonstrated that the seminiferous tubule structure in the Control and Inhibitor groups remained intact, with clearly defined layers of spermatogenic cells. The number and morphology of spermatozoa in the lumen were overall comparable to the normal baseline. In contrast, the PM2.5 exposure group exhibited significant histological damage: a marked reduction in sperm count within the tubules and a substantially increased proportion of abnormal spermatozoa, indicating that PM2.5 disrupts the spermatogenic microenvironment and impairs spermatogenesis. The PM2.5 + Inhibitor group showed a clear ameliorative trend compared to the PM2.5 exposure group: although a certain degree of reduced sperm count and increased abnormalities persisted, the number of intratubular spermatozoa was significantly higher than in the PM2.5 exposure group, and the number of abnormal sperm was reduced. This suggests that Caspase-1 inhibition exerts a protective effect against PM2.5-induced testicular injury (P < 0.05). This histological evidence provides a direct phenotypic foundation for subsequent validation of the inflammasome-pyroptosis axis mechanism.

As shown in Figure 3, Western blot analysis revealed no significant changes in the expression of the adaptor protein ASC, caspase-1, or GSDMD protein in the Inhibitor group compared to the Control group. The expression levels of all three key proteins were significantly elevated in both the PM2.5 group and the PM2.5 + Inhibitor group compared to the first two groups (p < 0.05). However, when comparing the PM2.5 + Inhibitor group to the PM2.5 group alone, the expression of all three proteins was significantly reduced (p < 0.05).

In the combined PM2.5 + Inhibitor intervention group, the protein expression levels showed a significant decline compared to the PM2.5 exposure group, yet remained markedly elevated relative to the Control and Inhibitor groups. This indicates that the Caspase-1 inhibitor effectively reverses the PM2.5-induced activation of the inflammatory inflammasome-pyroptosis axis. These findings form a consistent chain of causal evidence aligning with the aforementioned improvement in histopathology (P < 0.05).

As shown in Figure 6, heatmap analysis of the top 50 differentially expressed genes revealed a clear and consistent separation between the PM2.5-treated and control samples, with high intra-group similarity confirmed by Pearson correlation analysis. These results indicate robust transcriptomic alterations induced by PM2.5 exposure and good sample reproducibility.

Principal component analysis further demonstrated a distinct global shift in gene expression profiles between the two groups (Figure 7), suggesting that PM2.5 exposure is a dominant driver of transcriptional variation. Functional enrichment analysis of the DEGs showed that the most significantly enriched terms were predominantly related to immune and inflammatory responses, interferon signaling, and stress- or injury-associated biological processes.

To further identify key regulatory elements within these transcriptional changes, hub gene analysis was performed. As illustrated in Figure 8, the top-ranked hub genes, including STAT1, IRF9, GBP family members, IRGM1, and USP18, exhibited significant expression alterations and were mainly involved in interferon-mediated immune regulation and inflammatory amplification pathways.

Consistently, protein–protein interaction network analysis (Figure 9) revealed a highly interconnected core module enriched in STAT/IRF–GBP–IRGM-related signaling axes, highlighting a coordinated immune–inflammatory response network activated by PM2.5 exposure. Importantly, this transcriptional immune–stress network closely parallels the inflammasome–caspase-1–GSDMD pathway activation observed in the in vivo PM2.5 exposure and inhibitor intervention experiments, providing convergent evidence that PM2.5-induced testicular injury is associated with inflammasome-mediated inflammatory amplification.

As shown in Figure 6, the heatmap of the top 50 differentially expressed genes (DEGs) revealed a clear separation between PM2.5-exposed and control samples, with high intra-group consistency confirmed by Pearson correlation analysis, indicating robust PM2.5-induced transcriptional alterations.

Consistent with these findings, principal component analysis demonstrated distinct clustering of PM2.5-treated and control groups (Figure 7), with the first two principal components explaining the majority of variance. Functional enrichment of the DEGs showed significant involvement in defense response to virus, interferon signaling, and immune–inflammatory pathways, suggesting activation of stress-related immune programs following PM2.5 exposure.

Protein–protein interaction (PPI) network analysis identified key regulatory genes within this response. As shown in Figure 8, cytoHubba analysis highlighted ten hub genes (GBP7, GBP2, STAT1, IRF9, IGTP, IIGP1, IRGM1, USP18, PARP9, and PARP14), which were mainly associated with interferon-mediated immune regulation and inflammatory signaling. Corresponding bar plots further demonstrated their expression changes and enrichment in immune-related GO and KEGG terms.

Modular analysis using MCODE revealed a highly interconnected core module composed primarily of STAT/IRF–GBP–IRGM–USP18/PARP family members (Figure 9). This module suggests coordinated immune–inflammatory regulation and aligns with the activation of inflammasome-related pathways observed in subsequent in vivo experiments, supporting a mechanistic link between PM2.5 exposure and immune-mediated testicular injury.

Based on the GEO dataset GSE189187 (Control, n=3; PM2.5, n=3), and applying the pre-defined screening thresholds of P.Value < 0.05 and ∣log_2_FC∣ > 0.585 (approximately equivalent to FC > 1.5), a total of 217 differentially expressed genes (DEGs) were identified, consisting of 91 up-regulated and 126 down-regulated genes (see Figures 4 and 5). This finding indicates that PM2.5 exposure induces relatively widespread alterations in the transcriptional profile of testicular tissue, providing a candidate gene set for subsequent mechanistic and pathway analysis.

Functional enrichment analysis (Over-Representation Analysis, ORA, with P<0.05 / FDR correction) of the screened DEGs revealed that they were primarily enriched in biological processes related to immune-inflammation, response to stimuli/pathogen-associated molecules, as well as oxidative stress and tissue injury. Among the significant terms, the top five enriched entries (ranked by significance) included: *Defense response to virus* (P≈1.19×10^−13^ ; FDR≈ 1.68 × 10^−9^); *Acute lung injury* (P≈7.53×10^−11^; FDR≈5.32×10^−7^); *Contact dermatitis* (P≈2.54×10^−10^; FDR≈1.20 × 10^−6^); *Prostatic hyperplasia* (P≈1.01×10^−9^; FDR ≈ 3.56 × 10^−6^); and *Lung diseases* (P≈1.11×10^−9^; FDR ≈ 3.56 × 10^−6^). These terms collectively suggest that PM2.5 exposure-related transcriptional changes are not only associated with innate immunity/inflammatory amplification but are also statistically linked to stress-induced damage and pathological tissue processes.

To focus on the mechanism of the “inflammasome–caspase-1–GSDMD-mediated pyroptosis” axis, the intersection of the DEG_screen_all set and a pre-curated pyroptosis gene set was taken, yielding Pyroptosis_DEGs. Through this process, two differential genes directly overlapping with the pyroptosis gene set were identified: HMOX1, which was up-regulated (log_2_FC ≈ +2.150, P ≈ 4.60 × 10^−4^), and STAT1, which was down-regulated (log_2_FC≈ −1.301, P≈5.83×10^−4^). This result demonstrates that the public transcriptomic data indeed contain differential signals consistent with pyroptosis/inflammatory amplification-related networks. STAT1 (a hub of the immune/interferon axis) and HMOX1 (involved in stress/redox regulation) can serve as key focal points for subsequent network analysis and experimental validation. The presence of differential genes related to pyroptosis and inflammasomes in the public data provides transcriptional-level support for the mechanistic hypothesis.

This study utilized the candidate genes from the pyroptosis-focused analysis to construct a PPI network in STRING, which was then imported into Cytoscape for topological exploration. Network analysis revealed a densely interconnected structure among the candidate genes, suggesting their likely co-involvement in a shared immune-inflammatory/response network. The MCODE algorithm identified one highly connected core module (Module 1), containing genes such as *Hmox1*, *Stat4*, *Socs*, etc. ^[17,18]^. From a functional perspective, this module, characterized by interferon/antiviral response components (STAT/IRF, GBP, IFIs) and inflammatory signaling-related factors (IL-27/IL-12 axis, PARP family), aligns highly with the viral immune response terms identified in the ORA analysis.

Screening for hub genes using cytoHubba yielded the following Top 10hubgenes: GBP7, GBP2, STAT1, IRF9, IGTP, IIGP1, IRGM1, USP18, PARP9, and PARP14. Both network topological analyses (MCODE + cytoHubba) consistently indicated that the STAT1/IRF9-GBP-IRGM-USP18 axis forms the key regulatory center of this network ^[19]^. The altered inflammatory response network identified in the public transcriptomic data corroborates the activation of the NLRP3/caspase-1/GSDMD axis observed in our in-house animal experiments. Together, these findings support the mechanism that PM2.5 induces inflammasome-mediated pyroptotic-like damage in testicular tissue.

Consistent with the indications from the public data, in the SD rat intranasal exposure model (PM2.5 at 10 mg/kg for 1 month; n=6 per group), H&E staining results showed a significant reduction in sperm count and a marked increase in abnormal sperm within the seminiferous tubules of the PM2.5 group. These pathological alterations were partially reversed by administration of the caspase-1 inhibitor Z-YVAD-FMK (intranasal, 5 μg/mg). At the molecular level, Western blot analysis revealed significant up-regulation of ASC, caspase-1, and GSDMD in the PM2.5 group (approximately 7–8 fold), while their expression levels decreased to about 4–5 fold in the PM2.5 + inhibitor group. This suggests that caspase-1 inhibition effectively attenuates PM2.5-induced activation of the inflammasome-caspase-1-GSDMD axis. Collectively, these results demonstrate that the immune/stress network alterations identified by public transcriptomic screening and the reversible evidence of the inflammasome-pyroptosis axis *in vivo* corroborate each other. This provides multi-level support for the mechanistic framework wherein PM2.5 induces testicular injury and triggers pyroptosis-like inflammatory amplification by activating the NLRP3/caspase-1/GSDMD pathway.

## 5. Discussion

This study focused on the mechanism by which PM2.5 induces testicular pyroptosis via the NLRP3/Caspase-1 pathway, constructing an integrated evidence framework comprising an animal model, molecular pathways, validation with public transcriptomic data, and network hub gene analysis. First, H&E staining in the animal model clearly demonstrated that PM2.5 exposure leads to reduced sperm count and increased abnormal sperm within the seminiferous tubules. The partial reversal of these effects by the Caspase-1 inhibitor suggests the functional relevance of the inflammation-associated pyroptotic process. Second, Western blot results showed a concurrent, significant upregulation of ASC, Caspase-1, and GSDMD in the PM2.5 exposure group, while their expression markedly decreased in the PM2.5 + pyroptosis inhibitor combination group. This pattern establishes a molecular cascade consistent with the classical pyroptosis pathway: upstream inflammasome assembly → executioner enzyme → GSDMD-mediated pore formation, which constitutes the core framework of pyroptosis^[20]^.

To verify whether the inflammasome-pyroptosis axis is indeed activated by PM2.5, we examined the protein expression of ASC, Caspase-1, and GSDMD. Using the protein expression levels in the normal group as the baseline, the Caspase-1 inhibitor group exhibited levels similar to the normal group, indicating that the inhibitor itself did not induce abnormal activation of the pyroptotic inflammasome. In contrast, the expression of these key pyroptosis-related proteins was significantly elevated in the PM2.5 exposure group. This reveals an overall enhancement pattern aligned with the typical pyroptotic pathway: upstream inflammasome assembly (NLRP3/ASC) → executioner enzyme (caspase-1) → pore-forming executioner protein (GSDMD). The canonical pyroptosis pathway depends on caspase-1-mediated substrate cleavage and inflammatory amplification, providing a molecular mechanistic explanation for the tissue damage induced by PM2.5^[21]^.

Building upon this foundation, we introduced the public transcriptomic dataset GSE189187 for reproducible screening. This dataset originates from a GC-2spd cell model treated with PM2.5, and multi-omics studies have suggested that PM2.5 can trigger mitochondrial damage, energy metabolism disruption, and concomitant transcriptomic alterations ^[9]^. Our analysis revealed that enrichment results significantly pointed towards innate immunity/inflammation-related programs (terms like response to virus), indicating that PM2.5 can induce a transcriptional state of defenseinflammation amplification in cells. Further PPI network analysis uncovered modules and hub genes such as STAT/IRF-GBP. These gene clusters typically reflect interferon-driven immune activation and can synergistically amplify the inflammasome-pyroptosis axis, thereby explaining the observed tissue damage and enhanced pyroptotic signaling at the animal level ^[22]^.

The key value of this study lies in transforming the “correlative clues” from public transcriptomics into causal and reversible evidence from animal experiments, thereby more reliably supporting the pyroptosis mechanism hypothesis for PM2.5-induced testicular injury. Public data analysis showed that PM2.5 exposure-related differential genes are functionally concentrated in immune-inflammatory and stress-injury processes. Network topology further converged these findings onto a core innate immune response module represented by STAT/IRF–GBP–IRGM–USP18/PARP, suggesting that PM2.5 may exacerbate local tissue damage by mobilizing innate immunity and inflammatory amplification networks. Notably, HMOX1 and STAT1, identified through pyroptosis-related screening, represent oxidative stress regulation and immune signaling hubs, respectively. Together with the enrichment results and PPI modules, they point towards a molecular underpinning of “stress-inflammation coupling,” providing a plausible upstream environment for inflammasome activation. More importantly, in the animal model, PM2.5 exposure caused structural damage to seminiferous tubules and impaired sperm morphology. The Caspase-1 inhibitor Z-YVAD-FMK could partially restore sperm count, reduce abnormality rates, and significantly downregulate the expression levels of ASC, Caspase-1, and GSDMD. This suggests that PM2.5-related damage is not merely non-specific toxic accumulation but involves a targetable inflammasome-Caspase-1-GSDMD effector pathway. Thus, evidence from the public transcriptomic network and the reversible results of *in vivo* inhibitor intervention jointly support that PM2.5-induced testicular injury may form a “damage-inflammation-further damage” positive feedback loop through inflammasome-mediated pyroptotic-like inflammatory amplification. This provides a theoretical basis for subsequently targeting the pyroptosis axis for intervention^[9,23]^.

Integrating public transcriptomics and animal model intervention experiments, this study constructed a closed-loop evidence chain of public screening, network focus, and *in vivo* validation. In the GSE189187 dataset (Control n=3 vs. PM2.5 n=3), differentially expressed genes were functionally enriched primarily in immune-inflammatory responses and stress-injury related processes. Screening along the pyroptosis axis yielded key differential signals overlapping with the pyroptosis gene set (HMOX1 up-regulated, STAT1 down-regulated), suggesting that PM2.5 exposure is accompanied by transcriptional-level changes in inflammation amplification/cell death-related networks. Further STRING–Cytoscape network analysis revealed that the candidate genes form a highly interactive core module. MCODE Module 1 was enriched for immune response axes such as STAT/IRF–GBP–IRGM–PARP, and the Top 10 hub genes identified by cytoHubba (GBP7, GBP2, STAT1, IRF9, IGTP, IIGP1, IRGM1, USP18, PARP9, PARP14) highly overlapped with this module, indicating a stable central structure of this network^[19]^.

It must be noted that the public data originates from a mouse spermatocyte cell line, while our animal evidence comes from rat testicular tissue, representing different species and model levels. Therefore, we position the public transcriptomic data as a source for candidate network screening and trend replication, while considering the HE-stained sections, WB bands, and inhibitor intervention results from the processed experimental animal model as primary evidence for validating the pyroptosis mechanism. This combination of reproducible screening and causal verification can significantly enhance the credibility of the conclusions and better meets the expectation for a complete evidence chain required by the aforementioned mechanism^[24]^.

## Supplementary Material

This is a list of supplementary files associated with this preprint. Click to download.

• file.xlsx

## Figures and Tables

**Figure 1:**
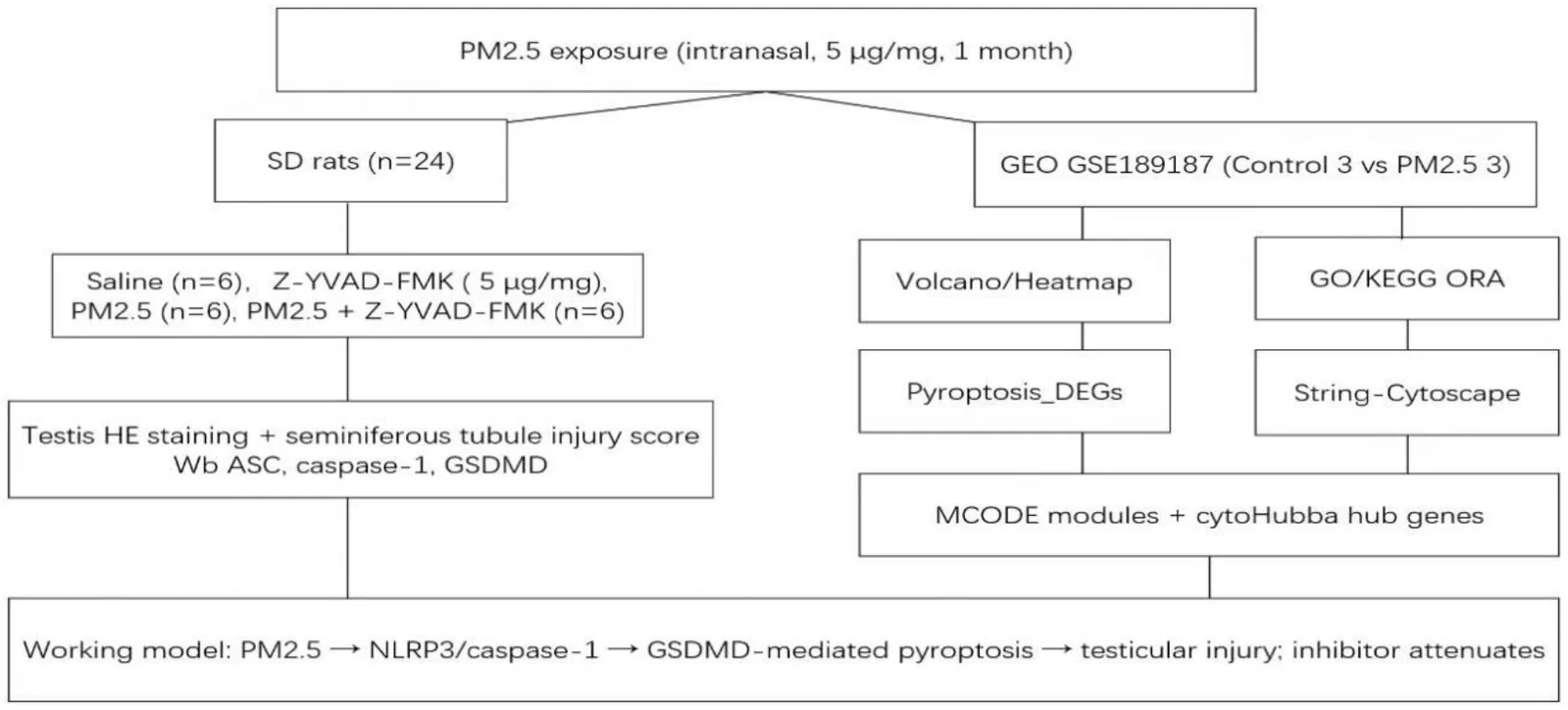
Study Design and Analysis Workflow

**Figure 2:**
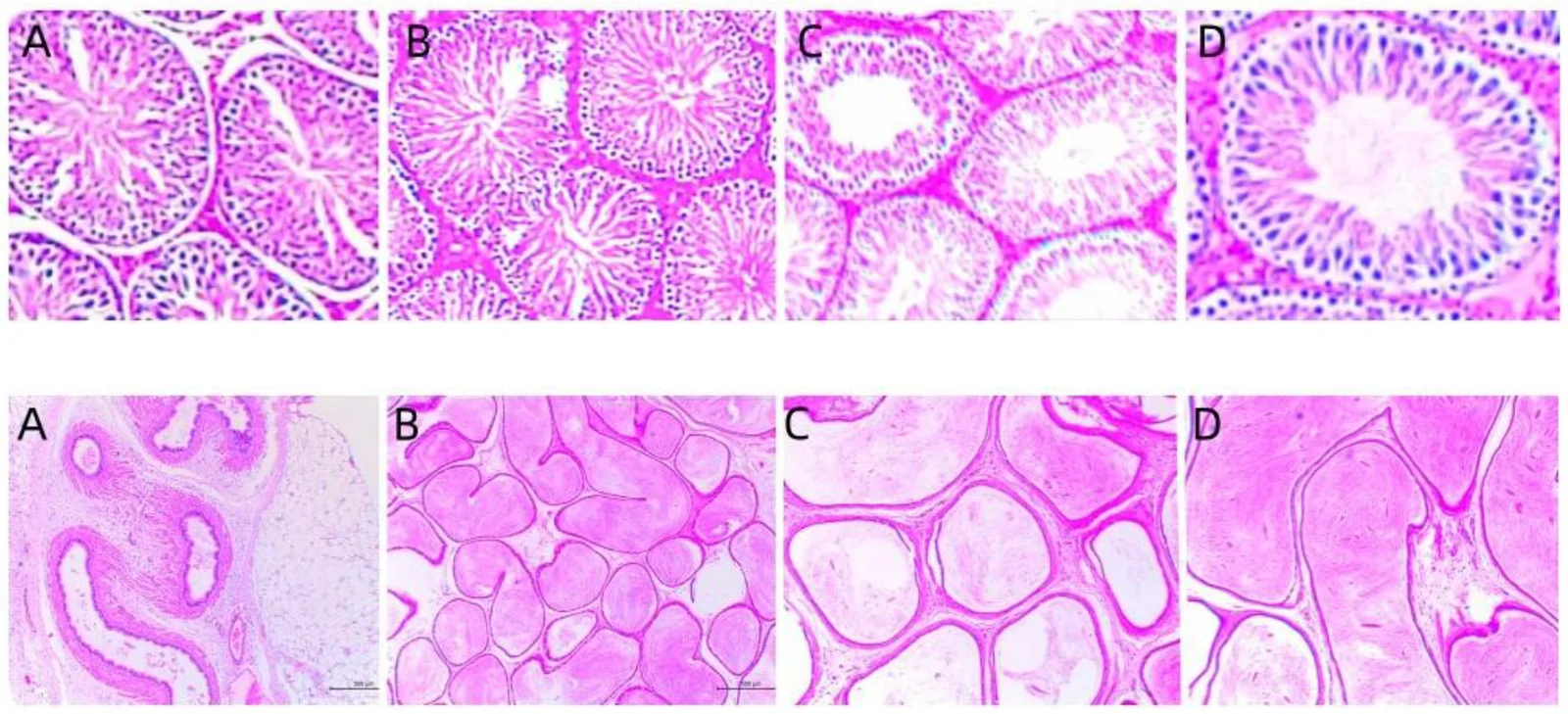
Morphological alterations in the testes and epididymis. A: Control group; B: Z-YVAD-FMK group; C: PM2.5 group; D: PM2.5 + Z-YVAD-FMK group. As shown: In Group A (A), numerous spermatozoa were observed within the seminiferous tubules, along with regularly arranged spermatogenic cells. Group B (B) showed no significant changes compared to Group A. In Group C (C), the seminiferous tubules exhibited substantial vacuolization accompanied by a marked reduction in sperm count. In Group D (D), sperm density was notably increased compared to Group C; however, relative to Groups A and B, sperm count remained significantly decreased, and the rate of sperm abnormalities was elevated.

**Figure 3:**
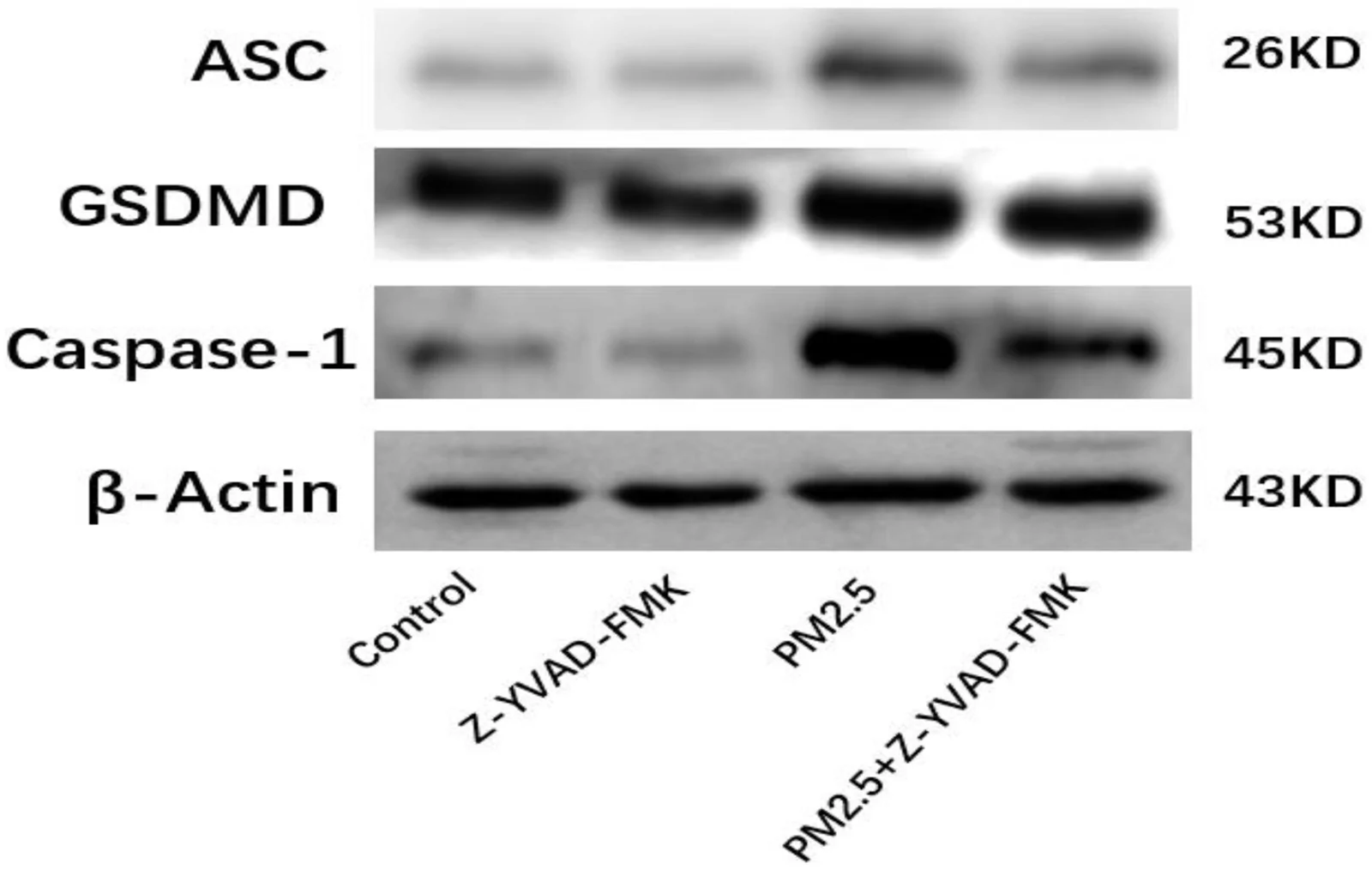
Bands of related proteins in the testis tissue.

**Figure 4:**
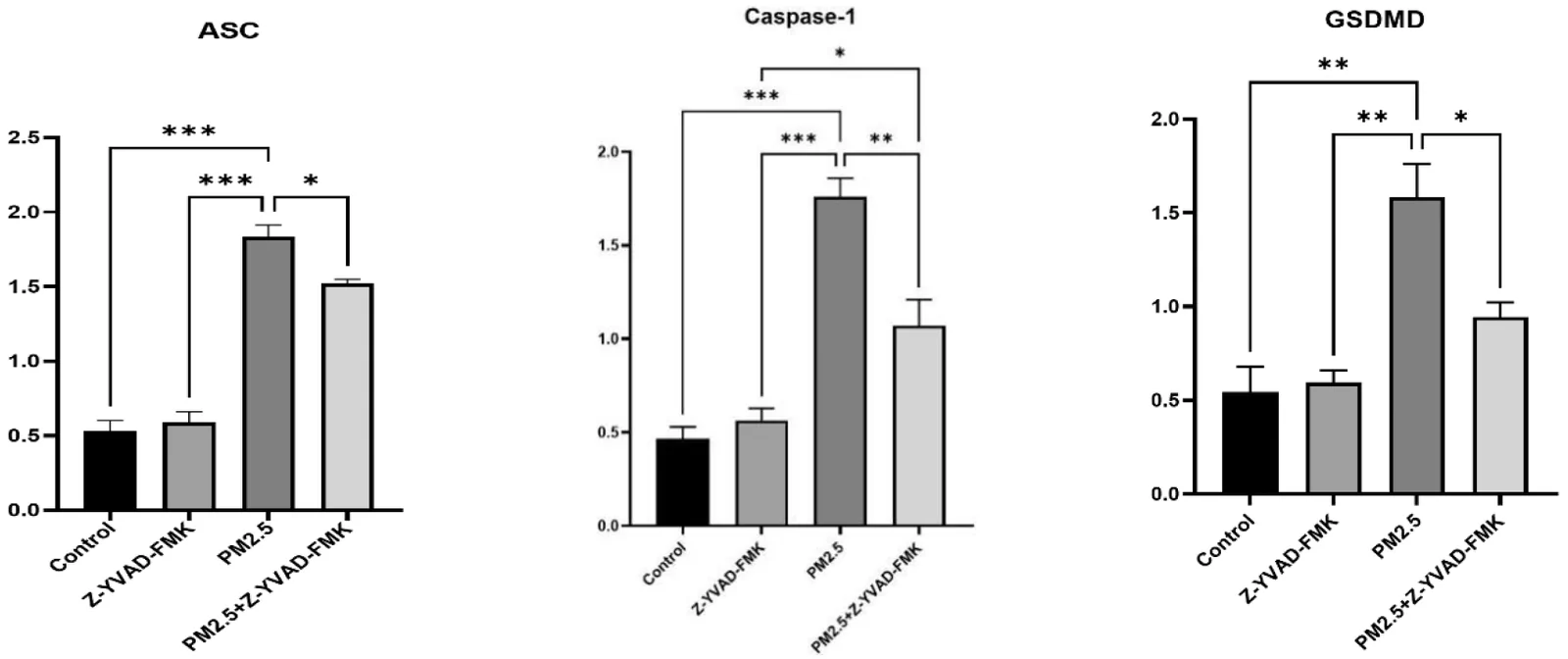
Quantification of protein expressions. Following PM2.5 exposure, the expression of ASC, GSDMD, and Caspase-1 was up-regulated, and this effect was reversed by intervention with the Caspase-1 inhibitor. Experiments were performed in triplicate, and data are presented as the mean ± standard deviation. * indicates P < 0.05, denoting a statistically significant difference between the compared groups.

**Figure 5:**
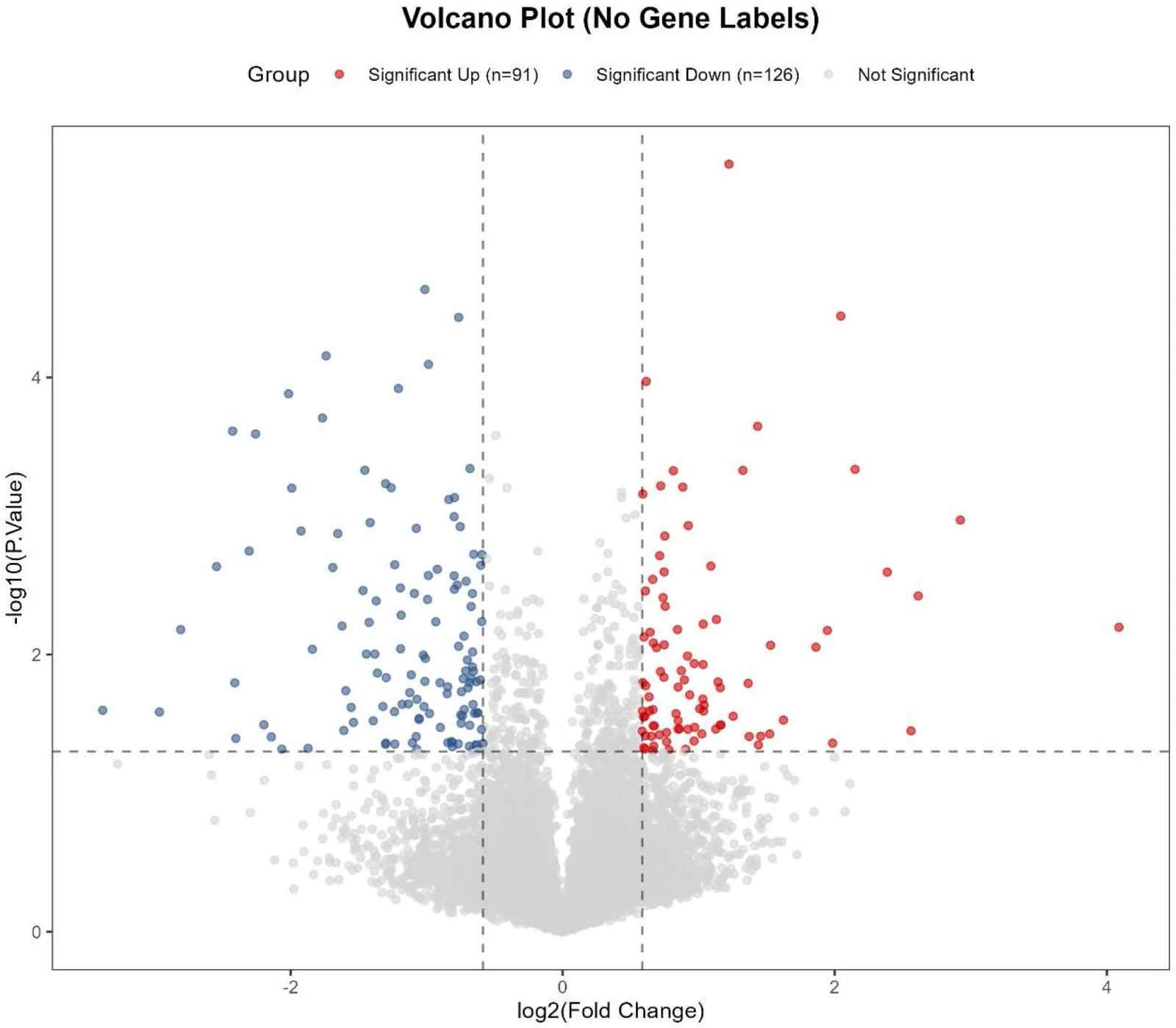
Volcano Plot of Differentially Expressed Genes (DEGs).

**Figure 6:**
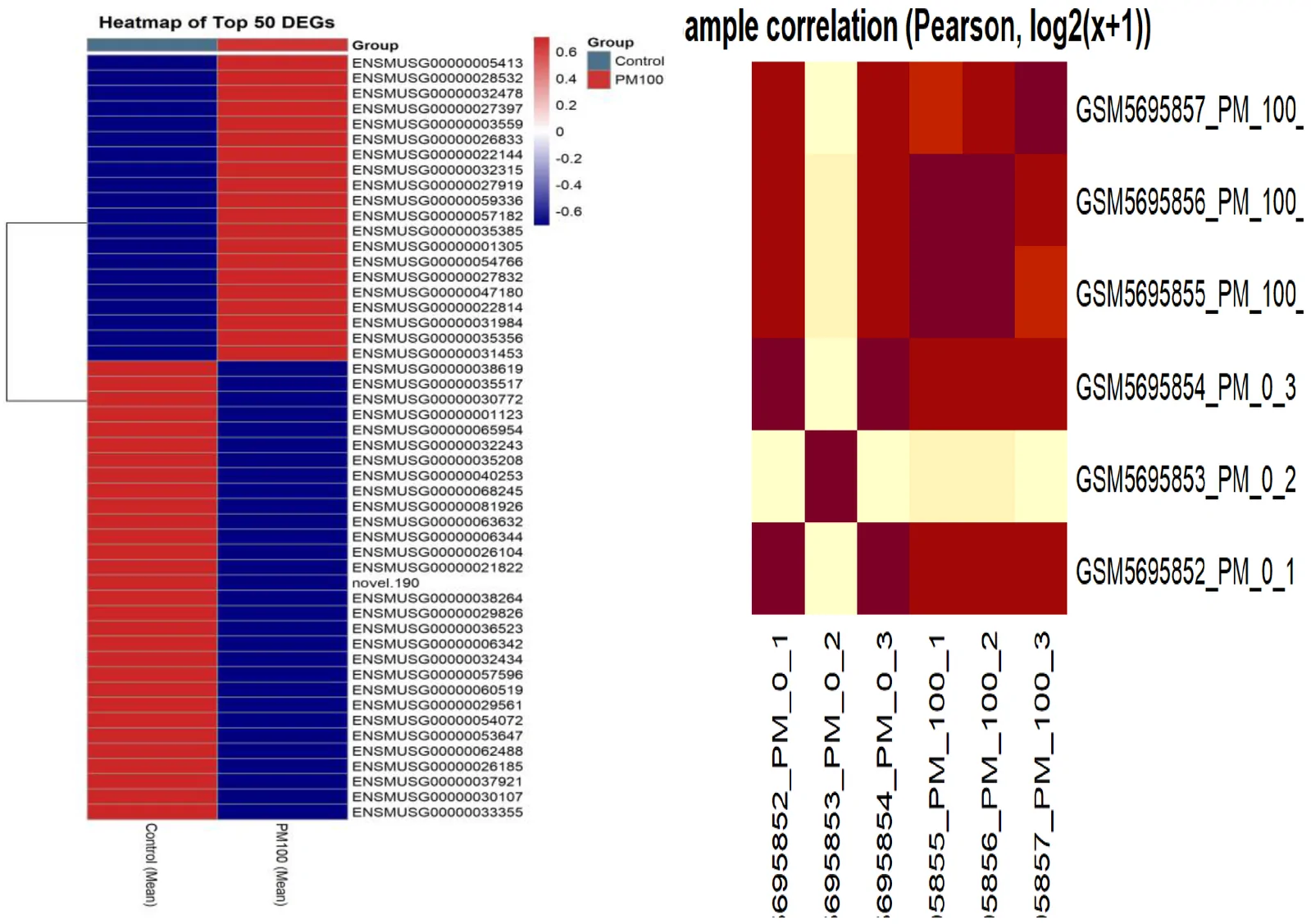
Expression Heatmap (Top 50 DEGs) and Sample Correlation Heatmap (Pearson)

**Figure 7:**
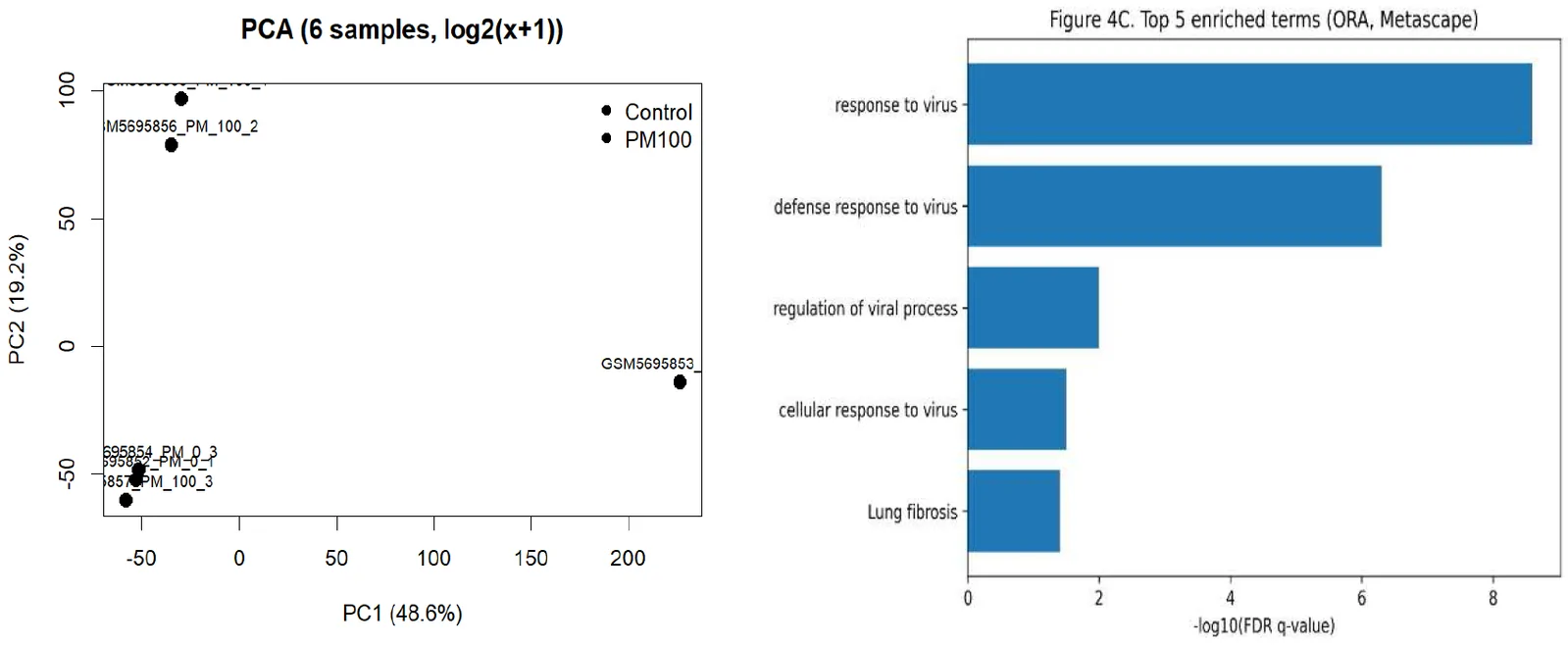
PCA scatter plot and bar chart of the top five enriched terms.

**Figure 8:**
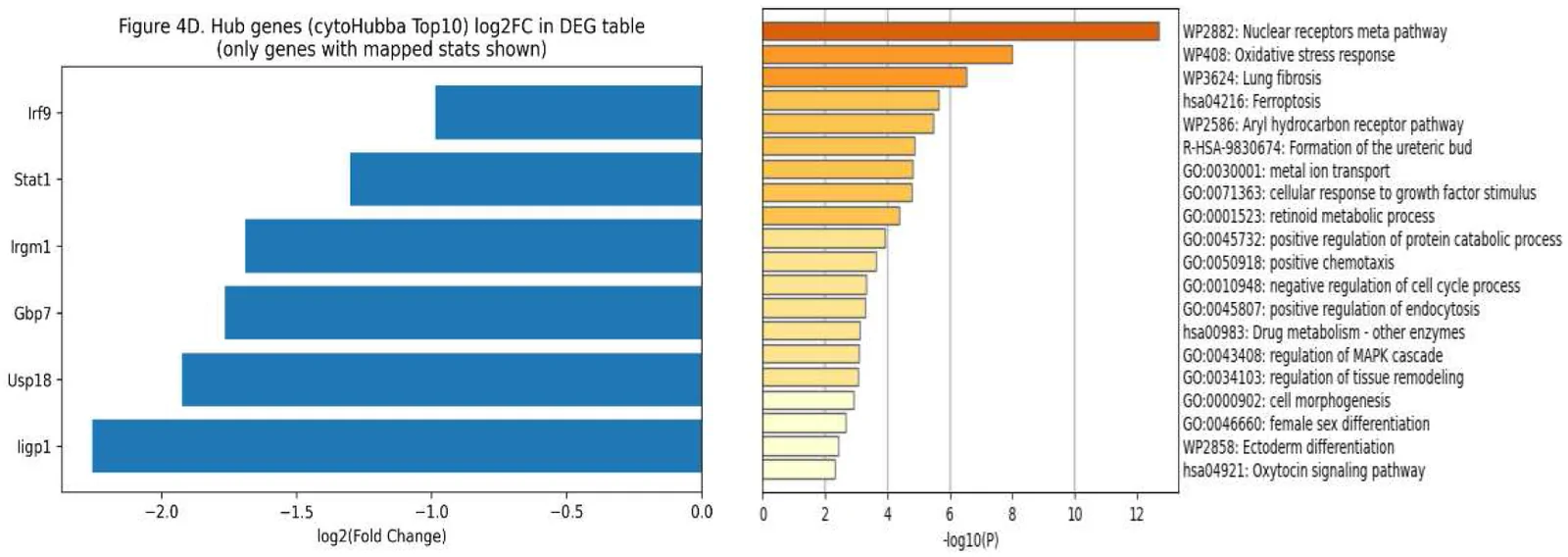
Bar chart of log2FC values for the Top 10 Hub Genes and bar charts of Pathway/GO Enrichment.

**Figure 9:**
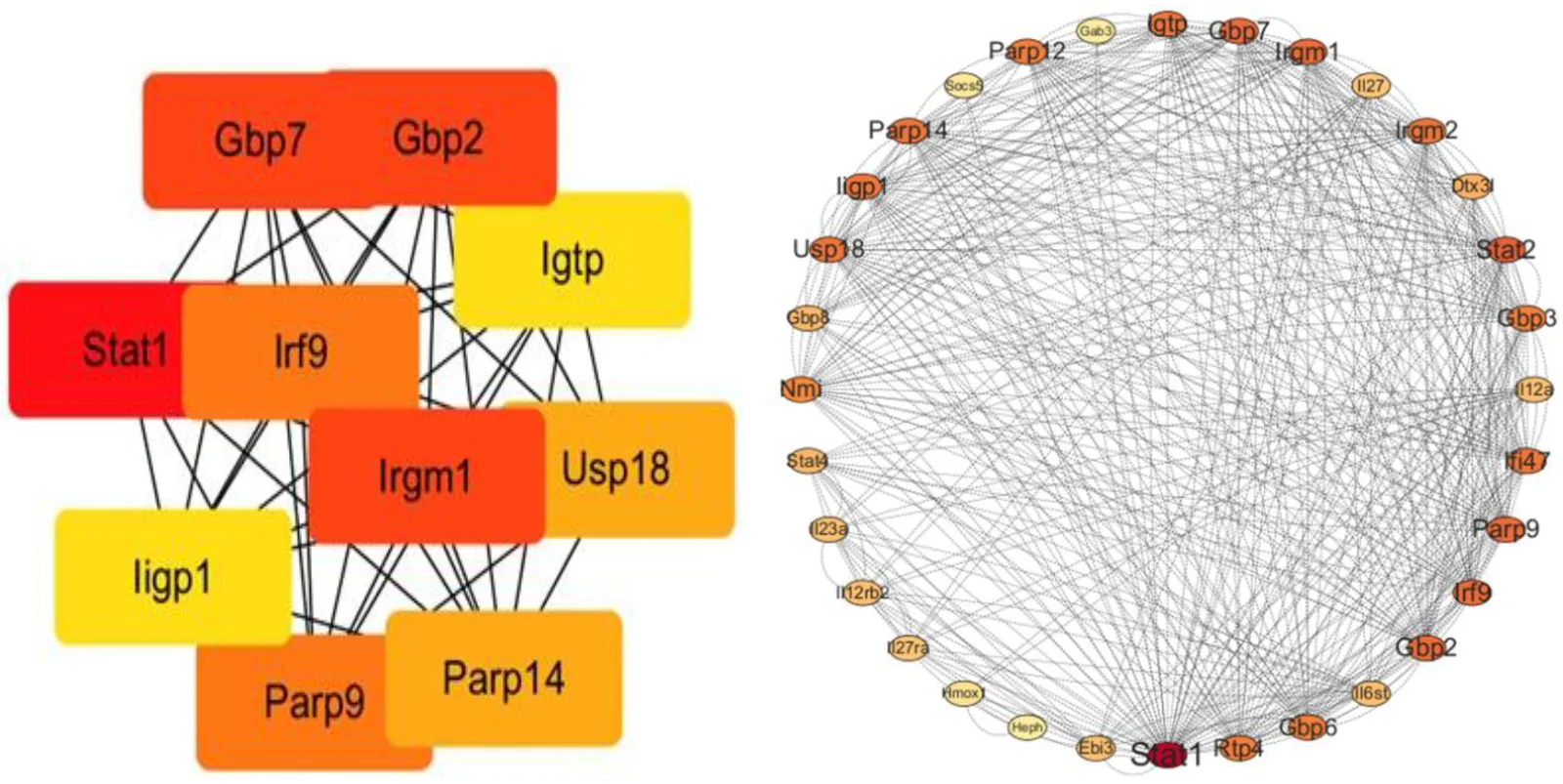
MCODE submodule hub gene cluster network and PPI (Protein-Protein Interaction) network.

**Table 1:** Body weight and absolute and relative organ weights of rats following PM2.5 exposure (mean ± SD).

Group	n	Body weight(g)	Testie (g)	Organ Index (%)	P value
Control	6	320.7±5.86	1.71±0.10	0.53±0.03	>0.05
Z-YVAD-FMK	6	322.7±6.66	1.68±0.12	0.52±0.03	>0.05
PM2.5	6	283.3±10.2	1.19±0.09	0.42±0.02	<0.01
PM2.5+Z-YVAD-FMK	6	306.7±2.08	1.55±0.08	0.50±0.02	<0.05

Note: Values are presented as the mean ± standard deviation (n=6). The P-value is derived from comparison with the Control group, with *p* < *0.05* considered statistically significant.

## Abbreviations

PM2.5: Particulate Matter 2.5
HE / H&E: Hematoxylin and Eosin
GEO: Gene Expression Omnibus
NCBI: National Center for Biotechnology Information
DEGs: Differentially Expressed Genes
ORA: Over-Representation Analysis
PPI: Protein-Protein Interaction
GO: Gene Ontology
KEGG: Kyoto Encyclopedia of Genes and Genomes
SD: Sprague-Dawley
ANOVA: Analysis of Variance
FDR: False Discovery Rate
FC: Fold Change
adj.P.Val: Adjusted P Value
NLRP3: NOD-like Receptor Family Pyrin Domain Containing 3
Caspase-1: Cysteine-containing Aspartate-specific Protease 1
GSDMD: Gasdermin D
ASC: Apoptosis-associated Speck-like protein containing a CARD
CARD: Caspase Activation and Recruitment Domain
IL-1β: Interleukin 1 Beta
IL-18: Interleukin 18
